# Supplementary material for: Artemisinin-resistant K13 mutations rewire Plasmodium falciparum’s intra-erythrocytic metabolic program to enhance survival
Source: Nat Commun. 2021 Jan 22;12:530. doi: 10.1038/s41467-020-20805-w (PMC7822823; doi:10.1038/s41467-020-20805-w)
Supplement: Supplementary file 1 — Supplementary Information [file 41467_2020_20805_MOESM1_ESM.pdf]

Supplementary Information

Artemisinin-resistant K13 mutations rewire *Plasmodium falciparum's* intra-erythrocytic metabolic program for survival

Sachel Mok, Barbara H. Stokes, Nina F. Gnädig, Leila S. Ross, Tomas Yeo, Chanaki Amaratunga, Erik Allman, Lev Solyakov, Andrew R. Bottrill, Jaishree Tripathi, Rick M. Fairhurst, Manuel Llinás, Zbynek Bozdech, Andrew B. Tobin, David A. Fidock

Table of contents

Supplementary Figure 1. Time-course transcriptome profiles and Spearman rank correlations of K13 mutant and wild-type Dd2 and Cam3.II lines. Related to Fig. 1, 2, 4b, 4c and Supplementary Data 1.

Supplementary Figure 2. Transcriptomic analyses of the developmental age estimation for Cam3.II parasites, the number of DE genes in K13 mutant vs. WT Dd2 parasites, and DE gene expression levels in the K13 mutants relative to WT isogenic parasites. Related to Fig. 2b, c.

Supplementary Figure 3. Differentially expressed proteins in Cam3.II^{C580Y} and Cam3.II^{R539T} mutants compared to Cam3.II^{WT} parasites at ring or trophozoite stages. Related to Fig. 1, 3 and Supplementary Data 3.

Supplementary Figure 4. K13 peptides and mass spectra identified by proteomics analysis of isobarically-tagged Cam3.II isogenic parasite lines. Related to Fig. 3.

Supplementary Figure 5. Detection of differentially expressed metabolites in the Cam3.II^{C580Y} mutant relative to Cam3.II^{WT} line. Related to Fig. 3c, 4a.

Supplementary Figure 6. Representative Giemsa images showing Cam3.II^{R539T} or Cam3.II^{WT} parasite morphologies following 6h treatment with 700nM DHA or 0.1% DMSO vehicle. Related to Fig. 4d.

Supplementary Figure 7. Frequency of differentially expressed genes and changes in RNA expression levels in the Dd2 or Cam3.II lines after 6h pulsed exposure to 700 nM DHA. Related to Fig. 4 and Supplementary Data 6.

Supplementary Figure 8. Functional enrichment analyses of genes differentially expressed in the DHA-treated $Dd2^{R539T}$ or $Dd2^{C580Y}$ mutants compared with $Dd2^{WT}$ across the 48h sampling time. Related to Fig. 4e and Supplementary Data 7.

Supplementary Figure 9. IC_{50} and IC_{90} fold shift values for 72h dose response assays and drug-pair isobologram analyses of Cam3.II and Dd2 K13 mutant vs. wild-type lines. Related to Fig. 5.

Supplementary Figure 10. Distribution of reporter ion intensities across the TMT channels for each of the four LC-MS/MS runs using $Cam3.II^{R539T}$, $Cam3.II^{C580Y}$ and $Cam3.II^{WT}$ K13 ring and trophozoite stage parasites in proteomic experiments. Related to Fig. 3.

Supplementary Figure 11. Flow cytometry pseudoplots showing the gating strategies for quantification of parasitemias in single and paired drug sensitivity assays.

Key Resources

Supplementary References

Supplementary Figure 1

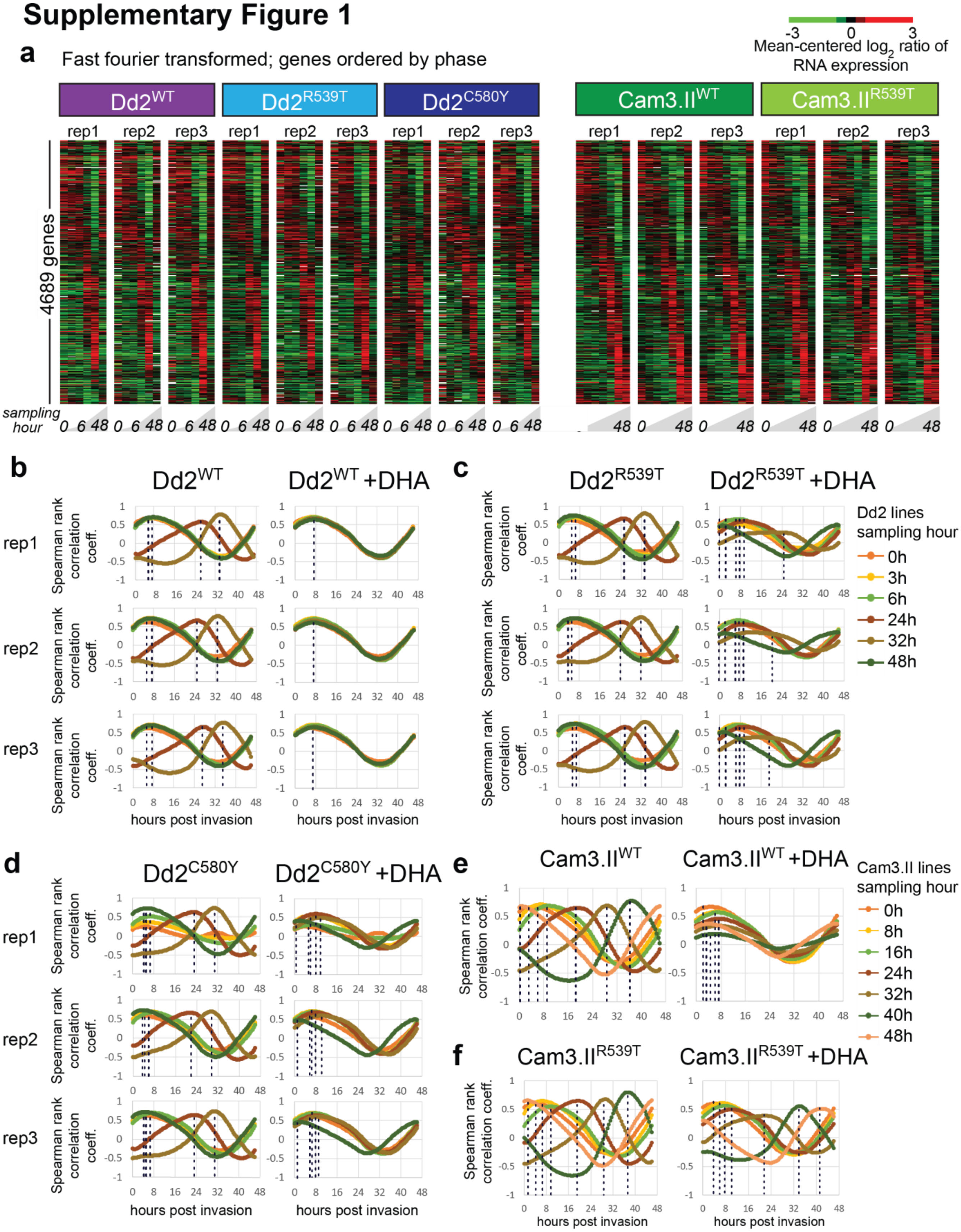

Supplementary Figure 1. Time-course transcriptome profiles and Spearman rank correlations of K13 mutant and wild-type Dd2 and Cam3.II lines. Related to Fig. 1, 2, 4b, 4c and Supplementary Data 1. a, Transcriptional profiles of K13 mutant and wild-type (WT) Dd2 and Cam3.II lines for 6-7 time points spanning the 48h asexual blood-stage cycle for each independent biological replicate experiment (N=3). Mean-centered $\log_2$ RNA expression ratios are shown for each of the 4,689 genes that passed the quality control in the samples. Ratios for each gene were calculated relative to the reference pool, containing a mixture of asexual blood stage RNA from 3D7 parasites. A $\log_2$ value of +3 or -3 indicates an 8-fold relative increase or decrease in transcript levels relative to the 3D7 reference pool, respectively. Genes were sorted by phase values derived from Fast Fourier transformations. To identify DE genes, we used non mean-centered data to avoid issues possibly arising from outliers. Our 0h time point data showed minimal variance between bio-reps, with a mean±SD correlation of 0.77±0.13 across lines. By comparison, when compared against the 24h time points, the mean correlation decreased to 0.21. **b-f**, Spearman rank correlation plots of time points sampled for the K13 WT **(b,e**) or mutant lines (**c,d,f**) compared against the Dd2 reference[1]. Parasites were exposed to 700 nM DHA or 0.1% DMSO vehicle control for 6h, starting with very early rings (0-3 hpi). Dd2 reference data were obtained from samples collected every 2h and $\log_2$ RNA expression values were imputed to give 96 time points at 30-minute intervals. For every time point sample, Spearman rank correlations of the $\log_2$ expression ratios were calculated between the sample and each of the 30-minute time points of the Dd2 reference for all ~5,000 genes. As an example, the 24h time points for $Dd2^{WT}$ have peaks that correspond to 25.0-27.5 hpi for the reference Dd2 data (shown as dashed lines) plotted on the X axis. The Dd2+DHA time points all showed an early ring-stage pattern, consistent with DHA treatment causing early rings to rapidly stall. In the presence of DHA, $Dd2^{R539T}$ parasites showed a developmental spread and loss of synchronicity from 24h onwards, indicative of parasites reinitiating growth at different times. Similar results were observed with DHA-treated $Cam3.II^{R539T}$ parasites. A description of these lines is provided in Table 1. We note that large-scale genome analysis by the Malaria Genomic Epidemiology Network[2] has uncovered 92 distinct K13 amino acid substitutions in clinical isolates. These mutations tend to cluster geographically and are almost always present as a single mutation per variant isolate. No copy number variations have been reported. Fewer than ten of these mutations have been confirmed to mediate *in vitro* resistance or to be associated with delayed parasite clearance[3].

Supplementary Figure 2

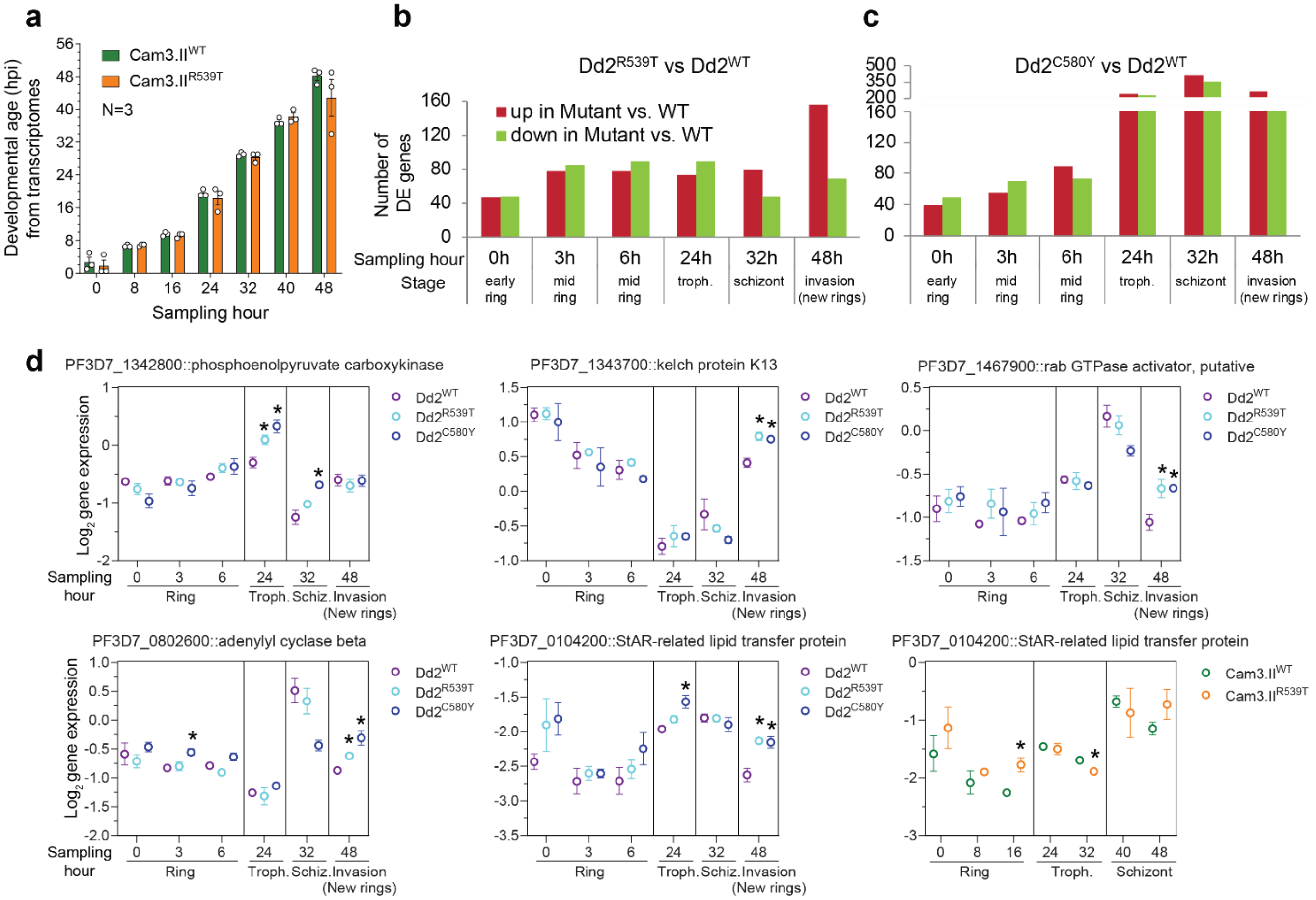

Supplementary Figure 2. Transcriptomic analyses of the developmental age estimation for Cam3.II parasites, the number of DE genes in K13 mutant vs. WT Dd2 parasites, and DE gene expression levels in the K13 mutants relative to WT isogenic parasites. Related to Fig. 2b, c. **a,** Transcriptomics-based age estimation for gene-edited K13 mutant and WT Cam3.II parasites, as determined by comparing against a Dd2 reference 48h intra-erythrocytic developmental cycle (IDC)[1]. Each time point was harvested on three independent occasions and data are presented as means ± SEM. **b-c**, Number of differentially expressed (DE) genes (red: up-regulated, green: down-regulated) in either **(b)** $Dd2^{R539T}$ or **(c)** $Dd2^{C580Y}$ lines as compared to the $Dd2^{WT}$ line at each sampling time point (ranging from early rings to late segmented schizonts) during the 48h IDC (*t*-test, *P*<0.05; N=3 independent experiments). **d,** RNA transcript levels for five DE genes that differed significantly between K13 R539T and C580Y mutants relative to WT in at least one sampling time point for Dd2 or Cam3.II parasites. Points and error bars represent the mean ± SEM of log_2 gene expression levels across three independent experiments for each parasite line; **P*<0.05, two-sided *t* tests. Calculated *P* values

per gene are: PF3D7_1342800 24h $Dd2^{R539T}$ vs $Dd2^{WT}$ P=0.03, $Dd2^{C580Y}$ vs $Dd2^{WT}$ P=0.01; 32h $Dd2^{C580Y}$ vs $Dd2^{WT}$ P=0.01; PF3D7_1343700 48h $Dd2^{R539T}$ vs $Dd2^{WT}$ P=0.01, $Dd2^{C580Y}$ vs $Dd2^{WT}$ P=0.009; PF3D7_1467900 48h $Dd2^{R539T}$ vs $Dd2^{WT}$ P=0.04, $Dd2^{C580Y}$ vs $Dd2^{WT}$ P=0.01; PF3D7_0802600 3h $Dd2^{C580Y}$ vs $Dd2^{WT}$ P=0.02, 48h $Dd2^{R539T}$ vs $Dd2^{WT}$ P=0.008, $Dd2^{C580Y}$ vs $Dd2^{WT}$ P=0.01; PF3D7_0104200 24h $Dd2^{C580Y}$ vs $Dd2^{WT}$ P=0.01, 48h $Dd2^{R539T}$ vs $Dd2^{WT}$ P=0.008, $Dd2^{C580Y}$ vs $Dd2^{WT}$ P=0.02; 16h $Cam3.II^{R539T}$ vs $Cam3.II^{WT}$ P=0.02; 32h $Cam3.II^{R539T}$ vs $Cam3.II^{WT}$ P=0.0001.

Supplementary Figure 3

a

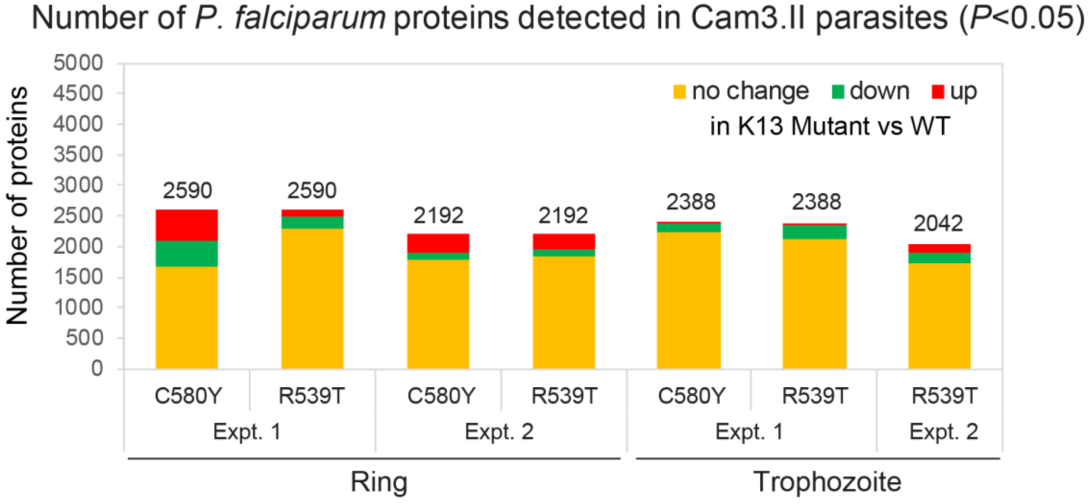

b

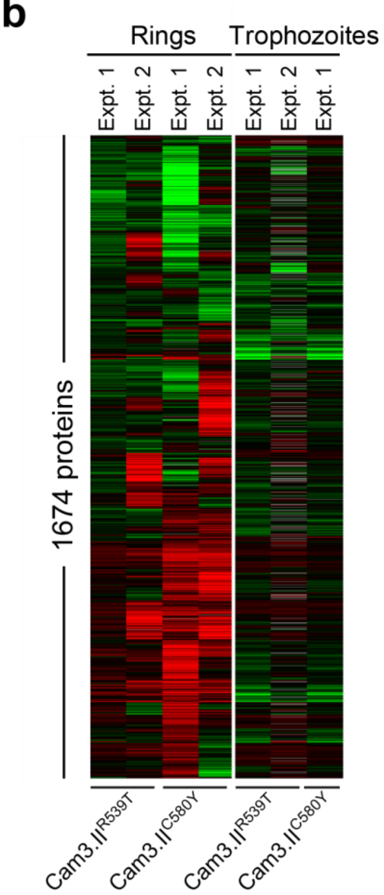

c

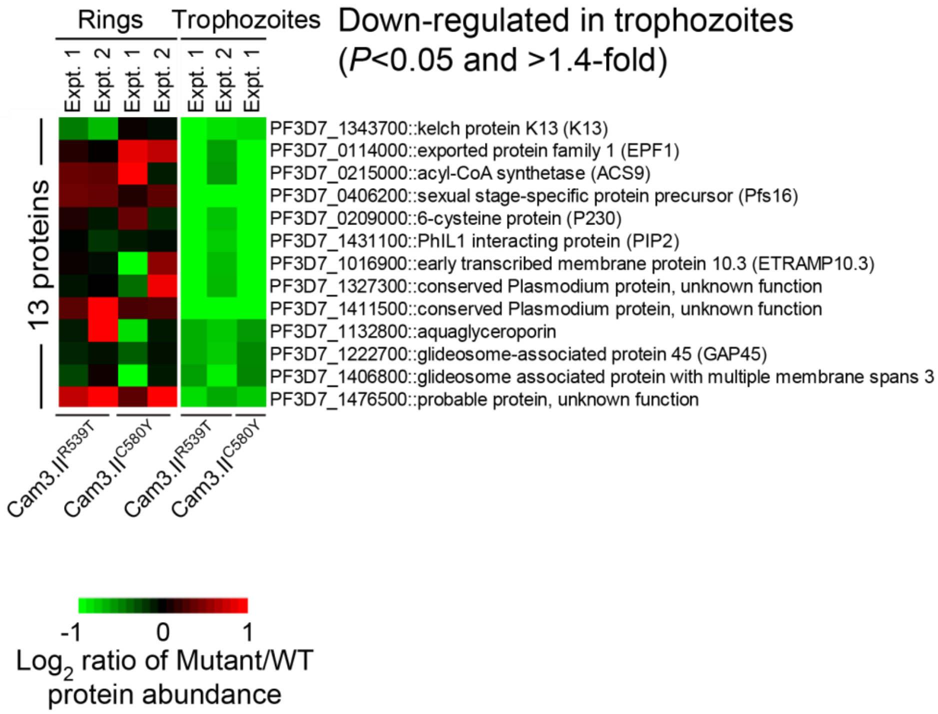

Supplementary Figure 3. Differentially expressed proteins in Cam3.II^{C580Y} and Cam3.II^{R539T} mutants compared to Cam3.II^{WT} parasites at ring or trophozoite stages. Related to Fig. 1, 3 and Supplementary Data 3. **a**, Numbers of differentially expressed (DE) proteins in Cam3.II K13 mutant relative to WT parasites across independent biological replicates sampled at ring (N=2) or trophozoite (N=1-2) stages. *t*-tests were used to compare $\log_2$ normalized peptide spectral intensities using peptide quantifications for each protein between strains in separate independent experiments. A cutoff of $P<0.05$ defined proteins that were significantly more or less abundant in the mutant lines. **b**, Heat map of hierarchically clustered $\log_2$ fold changes in protein levels for K13 mutant versus WT parasites, for 1,674 proteins that were detected in at least 6 of the 7 ring and trophozoite samples. The majority of DE proteins in K13 mutant parasites were observed at the ring stage. **c**, Heat map showing $\log_2$ fold changes for the 13 hierarchically clustered proteins that displayed significantly lower abundance in K13 mutant parasites as compared to WT parasites at the trophozoite stage. (two-sided *t* test, $P<0.05$ and >1.4-fold change). Notably, the K13 protein was less abundant in both Cam3.II K13 mutants at this stage, but only in Cam3.II^{R539T} at the ring stage.

Supplementary Figure 4

a

UL02183 run (Rings experiment 1)

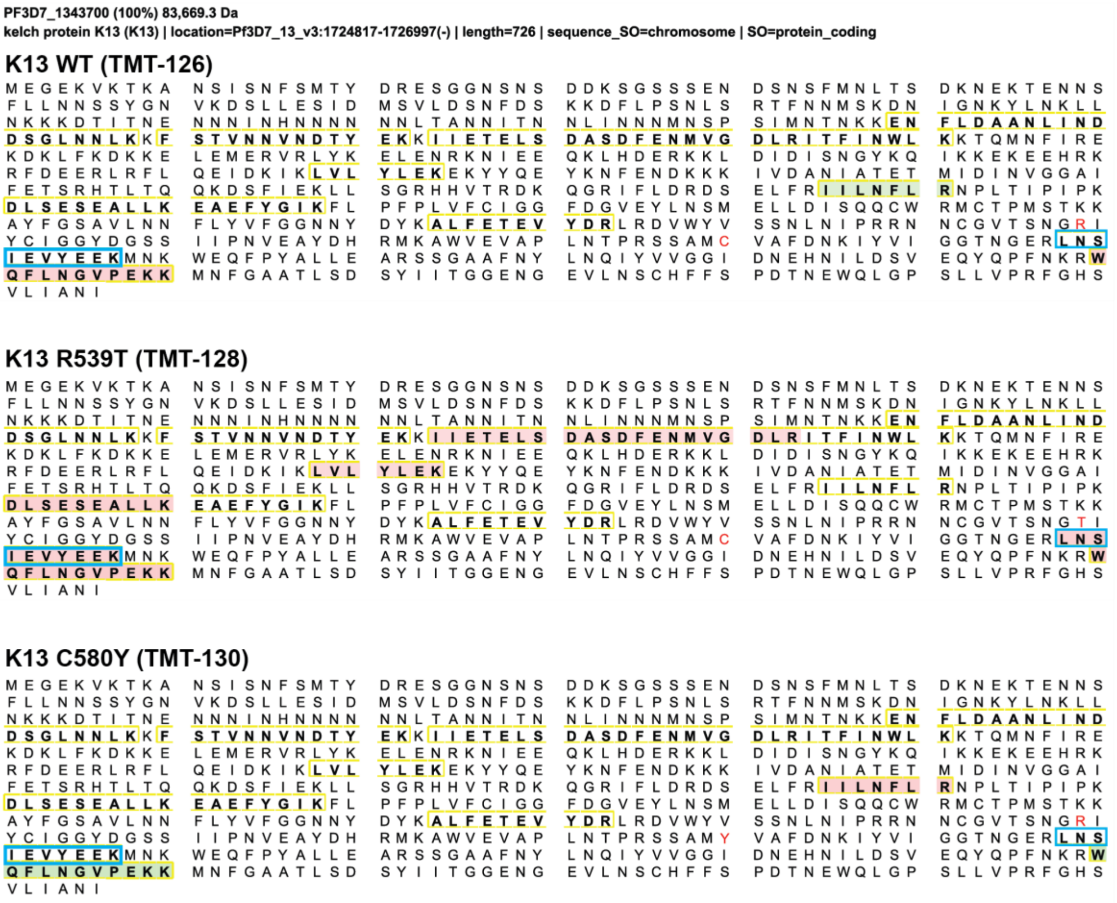

b

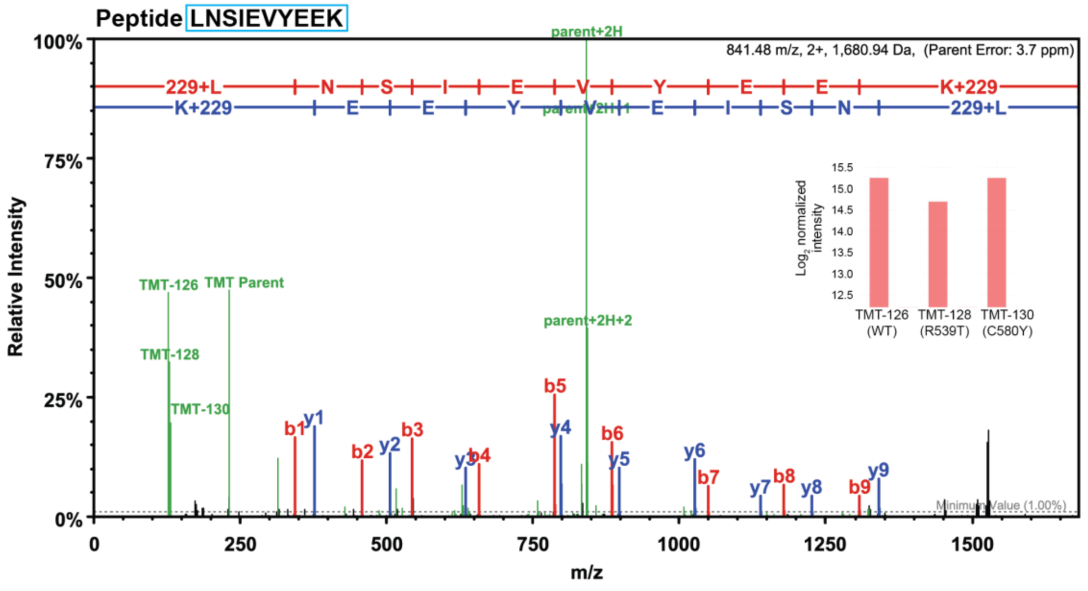

Supplementary Figure 4 (continued)

c UL02107 run (Rings experiment 2)

PF3D7_1343700 (100%) 83,669.3 Da
kelch protein K13 (K13) | location=Pf3D7_13_v3:1724817-1726997(-) | length=726 | sequence_SO=chromosome | SO=protein_coding

K13 WT (TMT-129)

```
MEGEKVKTKA NSISNFSMTY DRESGGNSNS DDKSGSSSEN DSNSFMNLTS DKNEKTENNS
FLLNNSSYGN VKDSLLESID MSVLDSNFDS KKDFLPSNLS RTFNNMSKDN IGNKYLNKLL
NKKKDTITNE NNNINHNNNN NNLTANNITN NLINNNMNSP SIMNTNKKEN FLDAANLIND
DSGLNNLKKF STVNNVNDTY EKKIIETELS DASDFENMVG DLRITFINWL KKTQMNFIRE
KDKLFKDKKE LEMERVRLYK ELENRKNIEE QKLHDERKKL DIDISNGYKQ IKKEKEEHRK
RFDEERLRFL QEIDKIKLVL YLEKEKYYQE YKNFENDKKK IVDANIATET MIDINVGGAI
FETSRHTLTQ QKDSFIEKLL SGRHHVTRDK QGRIFLDRDS ELFRIILNFL RNPLTIPIPK
DLSESEALLK EAEFYGIKFL PFPLVFCIGG FDGVEYLNSM ELLDISQQCW RMCTPMSTKK
AYFGSAVLNN FLYVFGGNNY DYKALFETEV YDRLRDVWYV SSNLNIPRRN NCGVTSNGRI
YCIGGYDGSS IIPNVEAYDH RMKAWVEVAP LNTPRSSAMC VAFDNKIYVI GGTNGERLNS
IEVYEEKMNK WEQFPYALLE ARSSGAAFNY LNQIYVVGGI DNEHNILDSV EQYQPFNKRW
QFLNGVPEKK MNFGAATLSD SYIITGGENG EVLNSCHFFS PDTNEWQLGP SLLVPRFGHS
VLIANI
```

K13 R539T (TMT-131)

```
MEGEKVKTKA NSISNFSMTY DRESGGNSNS DDKSGSSSEN DSNSFMNLTS DKNEKTENNS
FLLNNSSYGN VKDSLLESID MSVLDSNFDS KKDFLPSNLS RTFNNMSKDN IGNKYLNKLL
NKKKDTITNE NNNINHNNNN NNLTANNITN NLINNNMNSP SIMNTNKKEN FLDAANLIND
DSGLNNLKKF STVNNVNDTY EKKIIETELS DASDFENMVG DLRITFINWL KKTQMNFIRE
KDKLFKDKKE LEMERVRLYK ELENRKNIEE QKLHDERKKL DIDISNGYKQ IKKEKEEHRK
RFDEERLRFL QEIDKIKLVL YLEKEKYYQE YKNFENDKKK IVDANIATET MIDINVGGAI
FETSRHTLTQ QKDSFIEKLL SGRHHVTRDK QGRIFLDRDS ELFRIILNFL RNPLTIPIPK
DLSESEALLK EAEFYGIKFL PFPLVFCIGG FDGVEYLNSM ELLDISQQCW RMCTPMSTKK
AYFGSAVLNN FLYVFGGNNY DYKALFETEV YDRLRDVWYV SSNLNIPRRN NCGVTSNGTI
YCIGGYDGSS IIPNVEAYDH RMKAWVEVAP LNTPRSSAMC VAFDNKIYVI GGTNGERLNS
IEVYEEKMNK WEQFPYALLE ARSSGAAFNY LNQIYVVGGI DNEHNILDSV EQYQPFNKRW
QFLNGVPEKK MNFGAATLSD SYIITGGENG EVLNSCHFFS PDTNEWQLGP SLLVPRFGHS
VLIANI
```

K13 C580Y (TMT-130)

```
MEGEKVKTKA NSISNFSMTY DRESGGNSNS DDKSGSSSEN DSNSFMNLTS DKNEKTENNS
FLLNNSSYGN VKDSLLESID MSVLDSNFDS KKDFLPSNLS RTFNNMSKDN IGNKYLNKLL
NKKKDTITNE NNNINHNNNN NNLTANNITN NLINNNMNSP SIMNTNKKEN FLDAANLIND
DSGLNNLKKF STVNNVNDTY EKKIIETELS DASDFENMVG DLRITFINWL KKTQMNFIRE
KDKLFKDKKE LEMERVRLYK ELENRKNIEE QKLHDERKKL DIDISNGYKQ IKKEKEEHRK
RFDEERLRFL QEIDKIKLVL YLEKEKYYQE YKNFENDKKK IVDANIATET MIDINVGGAI
FETSRHTLTQ QKDSFIEKLL SGRHHVTRDK QGRIFLDRDS ELFRIILNFL RNPLTIPIPK
DLSESEALLK EAEFYGIKFL PFPLVFCIGG FDGVEYLNSM ELLDISQQCW RMCTPMSTKK
AYFGSAVLNN FLYVFGGNNY DYKALFETEV YDRLRDVWYV SSNLNIPRRN NCGVTSNGRI
YCIGGYDGSS IIPNVEAYDH RMKAWVEVAP LNTPRSSAMY VAFDNKIYVI GGTNGERLNS
IEVYEEKMNK WEQFPYALLE ARSSGAAFNY LNQIYVVGGI DNEHNILDSV EQYQPFNKRW
QFLNGVPEKK MNFGAATLSD SYIITGGENG EVLNSCHFFS PDTNEWQLGP SLLVPRFGHS
VLIANI
```

d **Peptide LNSIEVYEEK**

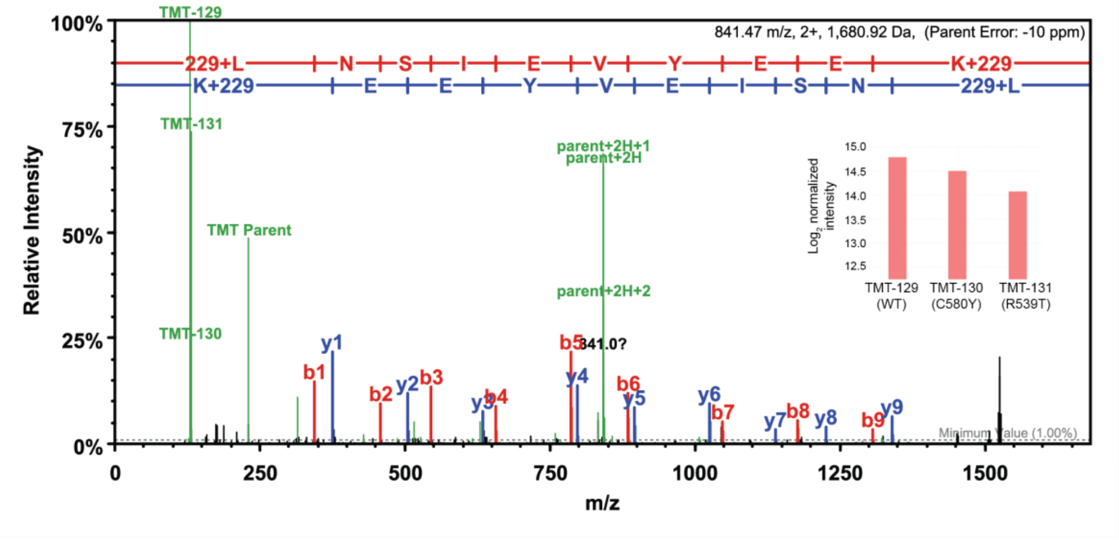

Supplementary Figure 4. K13 peptides and mass spectra identified by proteomics analysis of isobarically-tagged Cam3.II isogenic parasite lines. Related to Fig. 3. **a,** K13 protein sequence and peptides (yellow boxes) detected in the LC-MS/MS runs for ring-stage Cam3.II parasites harvested from one experiment that combined all three isogenic lines. The red and green highlights indicate the magnitude of the relative $\log_2$ normalized intensity, with red and green representing lower and higher abundance, respectively, of that peptide across samples when compared to the reference. **b,** Chromatograms showing the relative intensity spectra for the TMT-labeled K13 samples for a peptide sequence (blue box) that is adjacent to the C580Y mutation. Inset histogram shows the K13 peptide abundance after normalization of the global intensities. **c,d,** are graphical representations of the same results as shown in a,b and are from a second independent experiment.

Supplementary Figure 5

a

Table lists the number of technical replicate samples analyzed in each experimental run

Label	Stage	Condition: DMSO or DHA treated (in nM)	No. of samples for Cam3.II^{C580Y}	No. of samples for Cam3.II^{WT}
T1	Trophozoite	DMSO	3	--
		70	3	--
		350	3	--
T2	Trophozoite	DMSO	--	6
		70	--	3
		350	--	3
R1	Ring	DMSO	2	--
		350	1	--
		DMSO	--	2
		350	--	1
R2	Ring	DMSO	3	--
		70	3	--
		350	3	--
		DMSO	--	3
		70	--	3
		350	--	3
R3	Ring	DMSO	3	--
		70	3	--
		350	3	--
		DMSO	--	3
		70	--	3
		350	--	3

b

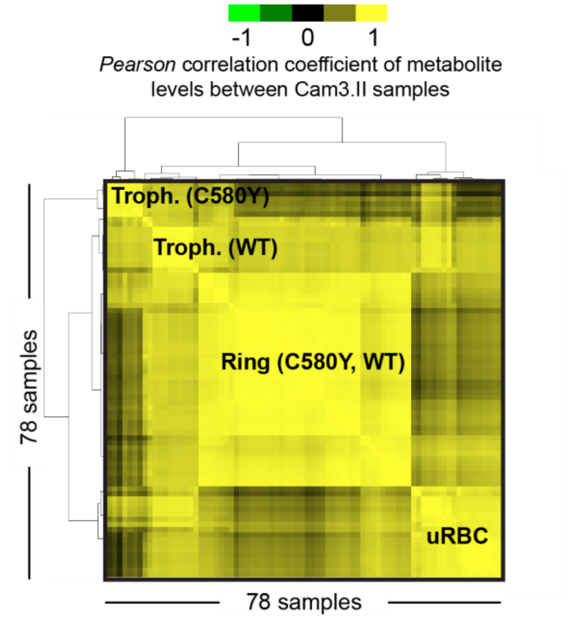

c

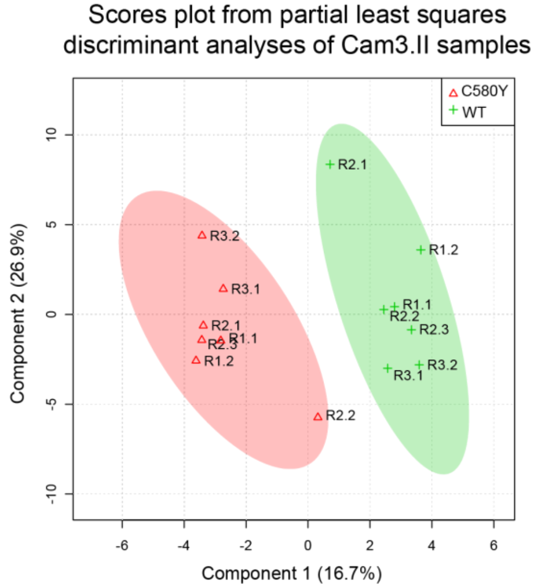

d

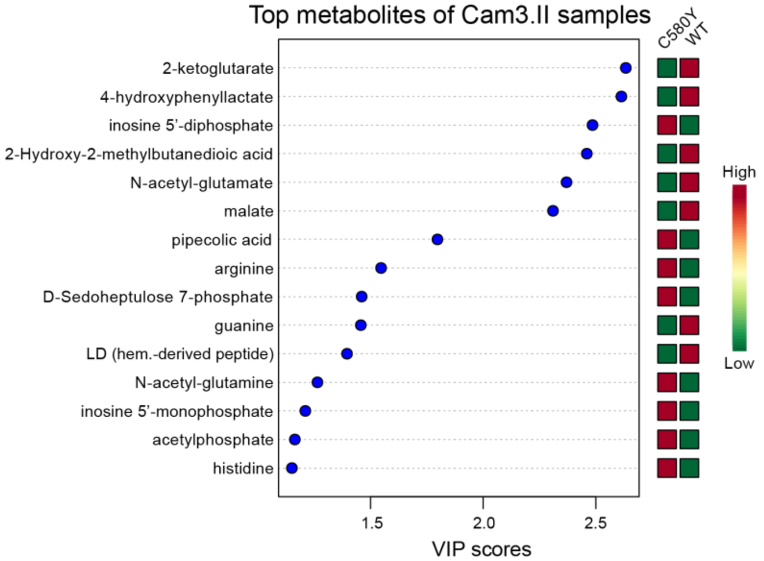

e

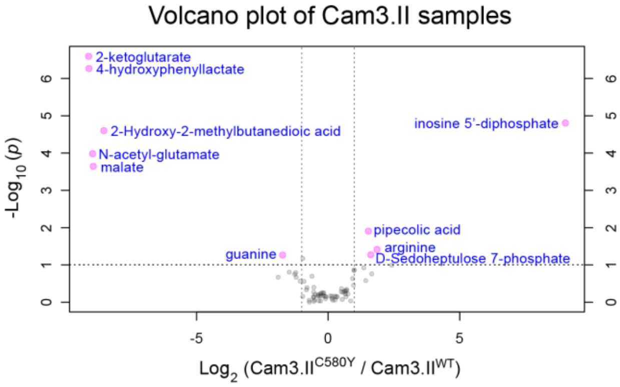

f

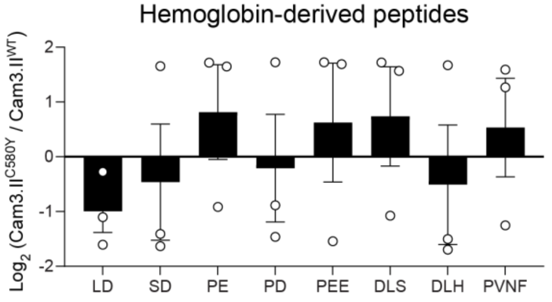

Supplementary Figure 5. Detection of differentially expressed metabolites in the Cam3.II^{C580Y} mutant relative to Cam3.II^{WT} line. Related to Fig. 3c and 4a. **a**, List of experimental samples collected across five LC-MS/MS assays with two trophozoite-stage (T1 and T2, prepared from Cam3.II^{C580Y} and Cam3.II^{WT} respectively) and three ring-stage experiments (R1, R2 and R3, prepared from Cam3.II^{C580Y} and Cam3.II^{WT} samples and ran in parallel) that gave a total of 96 metabolites. **b**, Sample to sample pair-wise Pearson correlation coefficients of metabolite levels determined from Cam3.II^{C580Y} and Cam3.II^{WT} ring and trophozoite samples. Results show clustering of samples by the parasite's developmental age, and all parasite samples clustered separately from uninfected RBC (uRBC) controls. **c**, Partial Least Squares Discriminant Analyses (PLS-DA) of the untreated Cam3.II^{C580Y} and Cam3.II^{WT} ring-stage samples (N, n = 3, 2-3) showing the samples distributed along the two main components of the scores plot. **d**, Key metabolites and their "variable importance in the projection" (VIP) scores identified from PLS-DA. The VIP score is measure of the contribution of the metabolite to the grouping and defined as the weighted sum of the squared correlations between the PLS-DA components and the metabolite. The colored boxes on the right indicate the relative levels of the metabolite in the Cam3.II^{C580Y} or Cam3.II^{WT} group. **e**, Volcano plot of the $-\log_{10}(P)$ values calculated from two-sided *t*-tests and the corresponding $\log_2$ fold change in metabolite levels for the untreated Cam3.II^{C580Y} vs. Cam3.II^{WT} ring-stage samples. Dotted line represents $P<0.1$ and >2-fold change. **f**, Log_2 fold change of hemoglobin-derived peptides levels in the untreated Cam3.II^{C580Y} vs. Cam3.II^{WT} ring-stage samples showing no marked differences in these peptide levels. Bar and dot plots represent the means ± SEM across three independent experiments and each experiment having two to three technical replicates.

Supplementary Figure 6

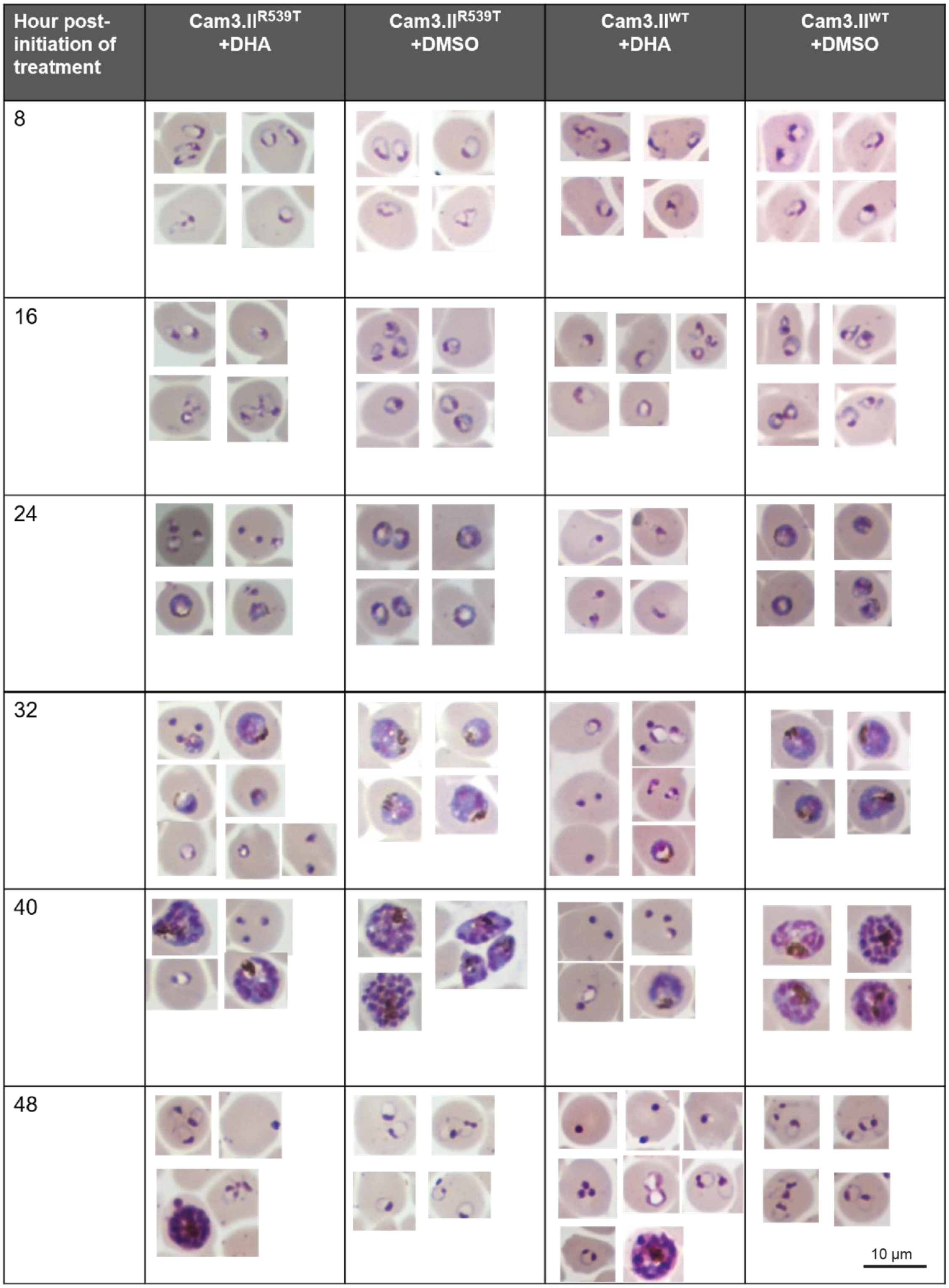

Supplementary Figure 6. Representative Giemsa images showing Cam3.II^{R539T} or Cam3.II^{WT} parasite morphologies following 6h treatment with 700nM DHA or 0.1% DMSO vehicle. Viable developing or pyknotic forms were observed following DHA treatment in the

mutant and wild-type lines. Microscopy-based visualization was performed for at least 5,000 cells per sample collected every 8h. We obtained similar results across three independent experimental repeats. Related to Fig. 4d.

Supplementary Figure 7

a

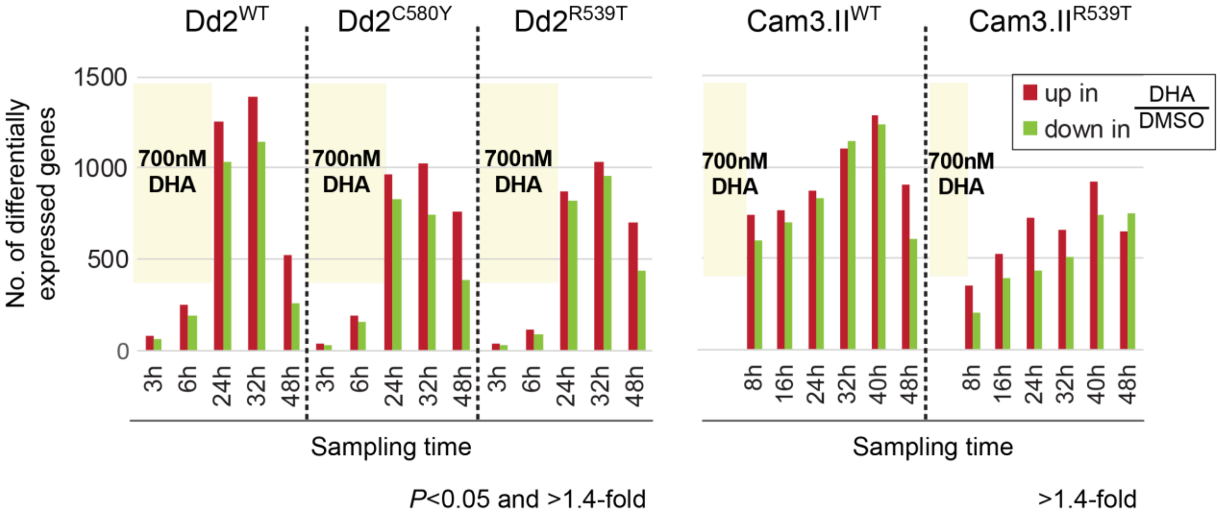

b

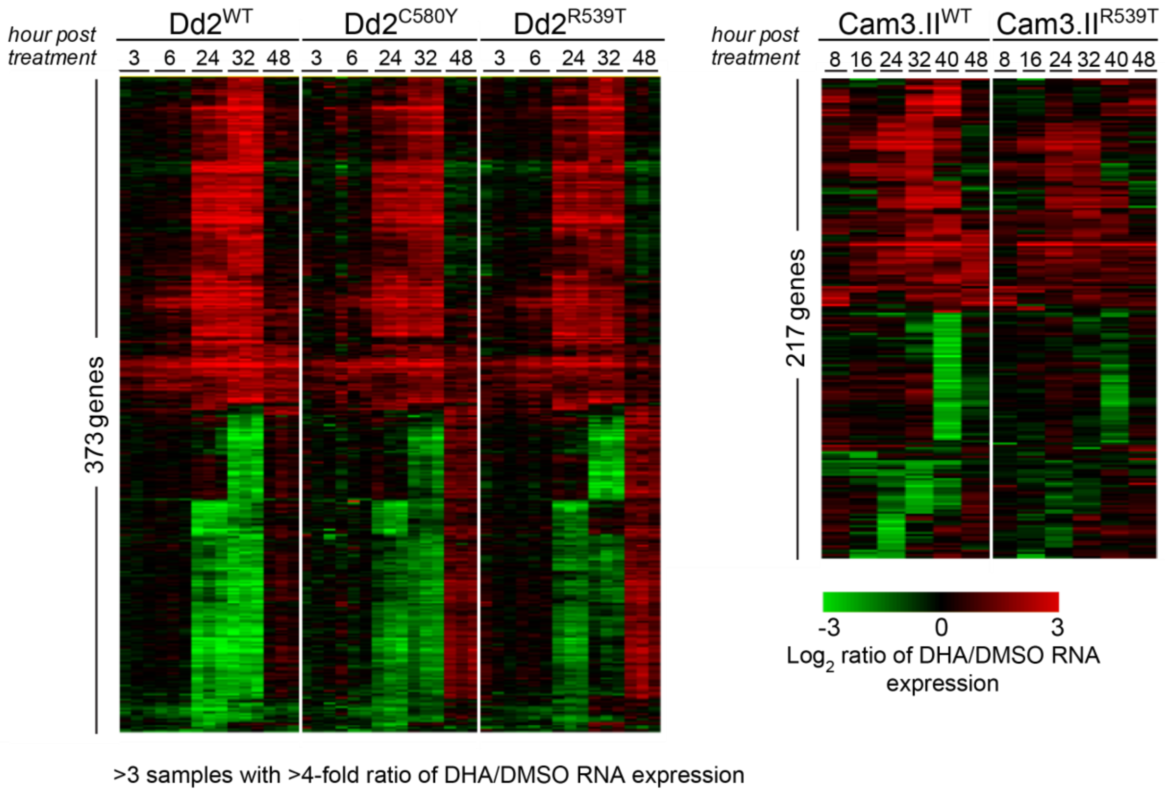

Supplementary Figure 7. Frequency of differentially expressed genes and changes in RNA expression levels in the Dd2 or Cam3.II lines after 6h pulsed exposures to 700 nM DHA. Related to Fig. 4 and Supplementary Data 6. **a**, Number of differentially expressed (DE) genes in DHA-treated relative to DMSO vehicle-treated parasites observed in the $Dd2^{WT}$, $Dd2^{C580Y}$ and $Dd2^{R539T}$ lines (N=3 independent experiments, two-sided *t* tests $P<0.05$ and >1.4-fold change) or in the $Cam3.II^{WT}$ and $Cam3.II^{R539T}$ (N=1, >1.4-fold change) lines. Fewer genes were DE in K13 mutants as compared to WT parasites. **b**, Heat maps of $\log_2$ fold changes for DE genes (hierarchically clustered) in K13 WT and mutant Dd2 (left) and Cam3.II (right) samples during the initial 6h of DHA exposure and after removal of DHA sampled up to 48h later. Shown are genes for which changes were present in at least three samples with greater than a four-fold difference (i.e. $\log_2$ of 2) in DHA-treated versus DMSO-treated parasites. The biggest transcriptional differences were observed at 24h and 32h post initiation of treatment (i.e. after removal of DHA) and changes were more pronounced in both Dd2 and Cam3.II K13 WT parasites compared to their respective mutant lines.

Supplementary Figure 8

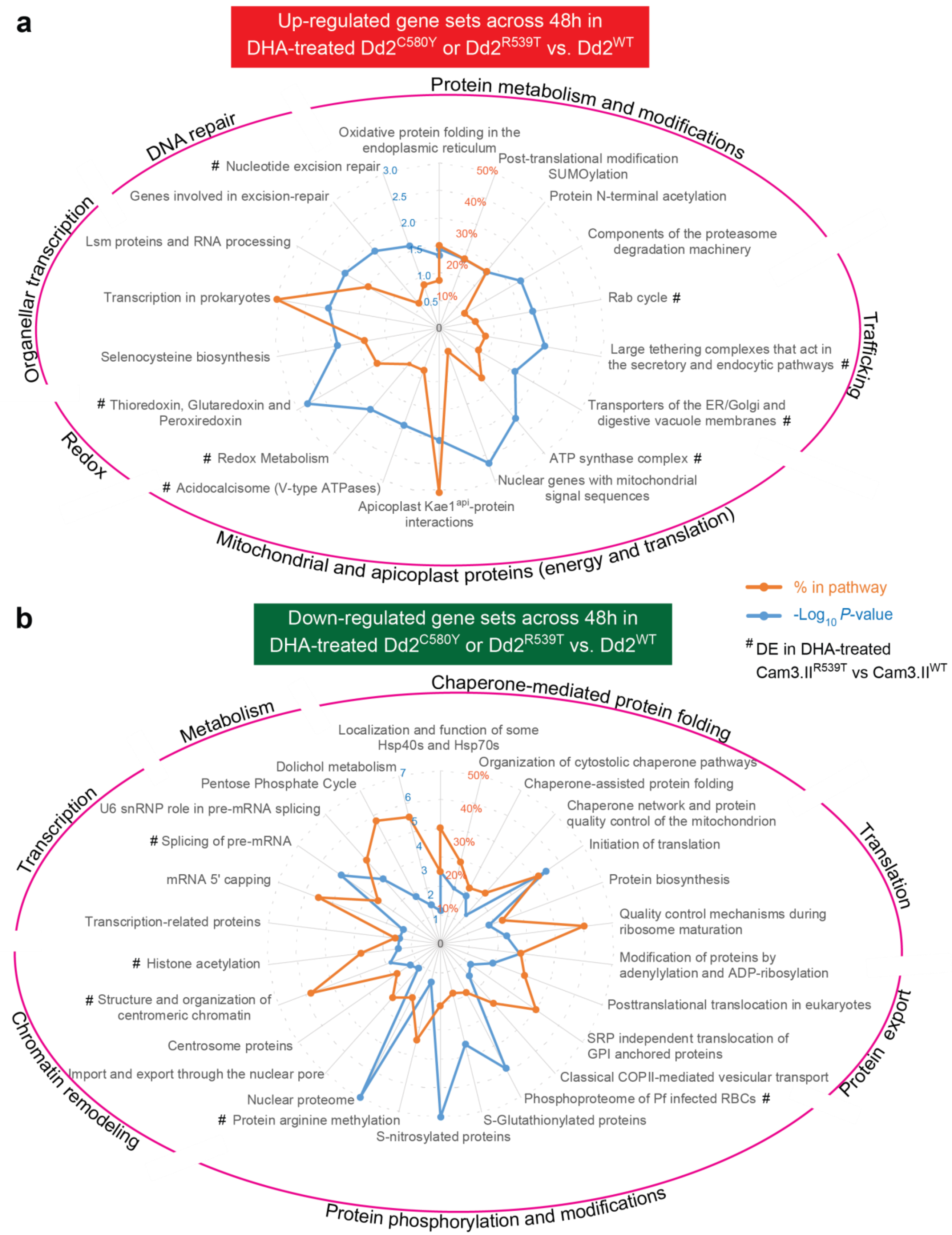

Supplementary Figure 8. Functional enrichment analyses of genes differentially expressed in the DHA-treated Dd2^{R539T} or Dd2^{C580Y} mutants compared with Dd2^{WT} across the 48h sampling time. Related to Fig. 4e and Supplementary Data 7. **a-b**, Functional pathways associated with genes that were significantly up- or down-regulated in the DHA-treated Dd2^{R539T} or Dd2^{C580Y} mutants compared with Dd2^{WT} and relative to their respective DMSO controls and categorized by the major cellular processes that these pathways belong to. Shown are the % of DE genes belonging to each pathway and the $-\log_{10}$ *P*-value of the hypergeometric test for over-enrichment of the DE genes in that pathway. Gene sets that overlapped with the similar analyses performed on Cam3.II^{R539T} vs. Cam3.II^{WT} lines is depicted with #. Most of the common up-regulated pathways in the DHA-treated Dd2 and Cam3.II K13 mutants are related to trafficking, organellar (mitochondria and apicoplast) functions, redox, and DNA repair that may be essential for the survival of the resistant mutants post DHA exposure.

Supplementary Figure 9

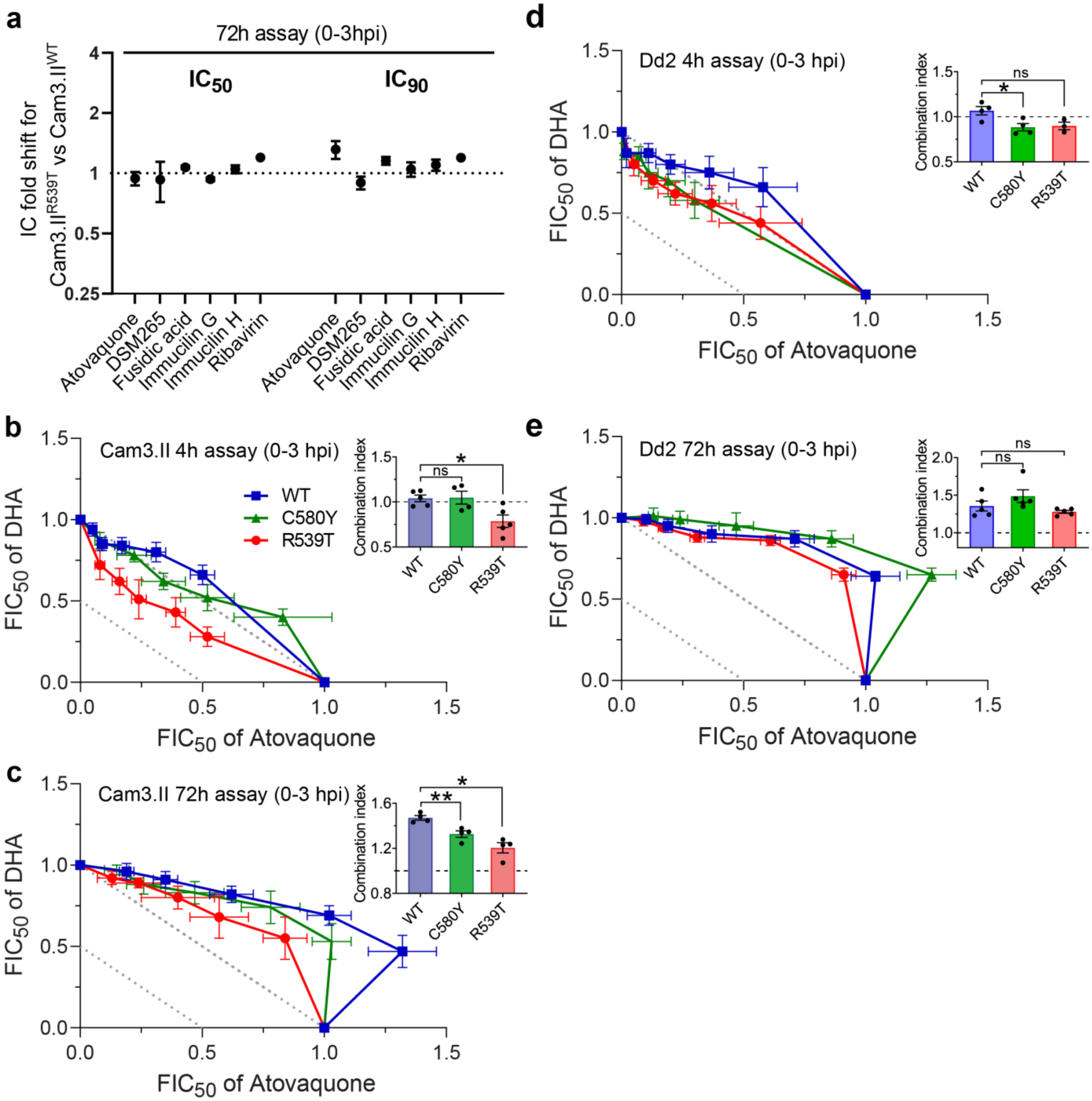

Supplementary Figure 9. IC_{50} and IC_{90} fold shift values for 72h dose-response assays and drug-pair isobologram analyses of Cam3.II and Dd2 K13 mutant vs. wild-type lines. Related to Fig. 5. **a**, IC_{50} and IC_{90} fold shifts for Cam3.II^{R539T} vs. Cam3.II^{WT} 0-3 hpi early rings exposed for 72h to a panel of inhibitors targeting mitochondrial processes (atovaquone, DSM265, fusidic acid) or purine metabolism (immucilin G and H, ribavirin). Shown are mean ± SEM values from two to four independent experiments with technical duplicates, except for ribavirin that was assayed once. Individual IC_{50} and IC_{90} data are provided in Supplementary

Data 8. **b-e**, Isobolograms showing the fractional IC_{50} (FIC_{50}) values of DHA + atovaquone combination pairs tested at fixed molar ratios of their individual IC_{50} values, comprising 1:0, 4:1, 2:1, 1:1, 1:2, 1:4, and 0:1 ratios of atovaquone to DHA. Assays were conducted on K13 C580Y or R539T mutants or WT parasites generated in either the Cam3.II or Dd2 backgrounds. Synchronized 0-3 hpi early ring-stage parasites were pulsed with drugs for 4h (**b,d**) or were continuously exposed for 72h (**c,e)**. FIC_{50} values are shown as means ± SEM derived from 3-5 biological repeats with technical duplicates. The dashed line at x=y=1 indicates additivity between the two compounds (when the sum FIC_{50}, also known as the Combination Index, equal to 1). Points lying substantially above the dashed line are indicative of an antagonistic interaction, whereas points lying substantially below the line indicate synergy. Combination Index insets depict the average of the sum of the FIC_{50} values calculated for each experiment across the range of dual-drug combination ratios. The DHA and atovaquone combination applied for 4h was synergistic in killing Cam3.II^{R539T} rings, unlike Cam3.II^{WT} which showed an additive effect. Interactions between these two drugs differed in 72h assays based on their *k13* sequence and genetic background. *$P<0.05$ and **$P<0.01$ (paired two-sided *t* tests); ns, not significant. For panel **b**, Cam3.II^{R539T} vs Cam3.II^{WT} yielded a *P* value of 0.015. For panel **c**, Cam3.II^{C580Y} vs Cam3.II^{WT} yielded a *P* value of 0.0091. Cam3.II^{R539T} vs Cam3.II^{WT} yielded a *P* value of 0.01. For panel **d**, Dd2^{C580Y} vs Dd2^{WT} yielded a *P* value of 0.02.

Supplementary Figure 10

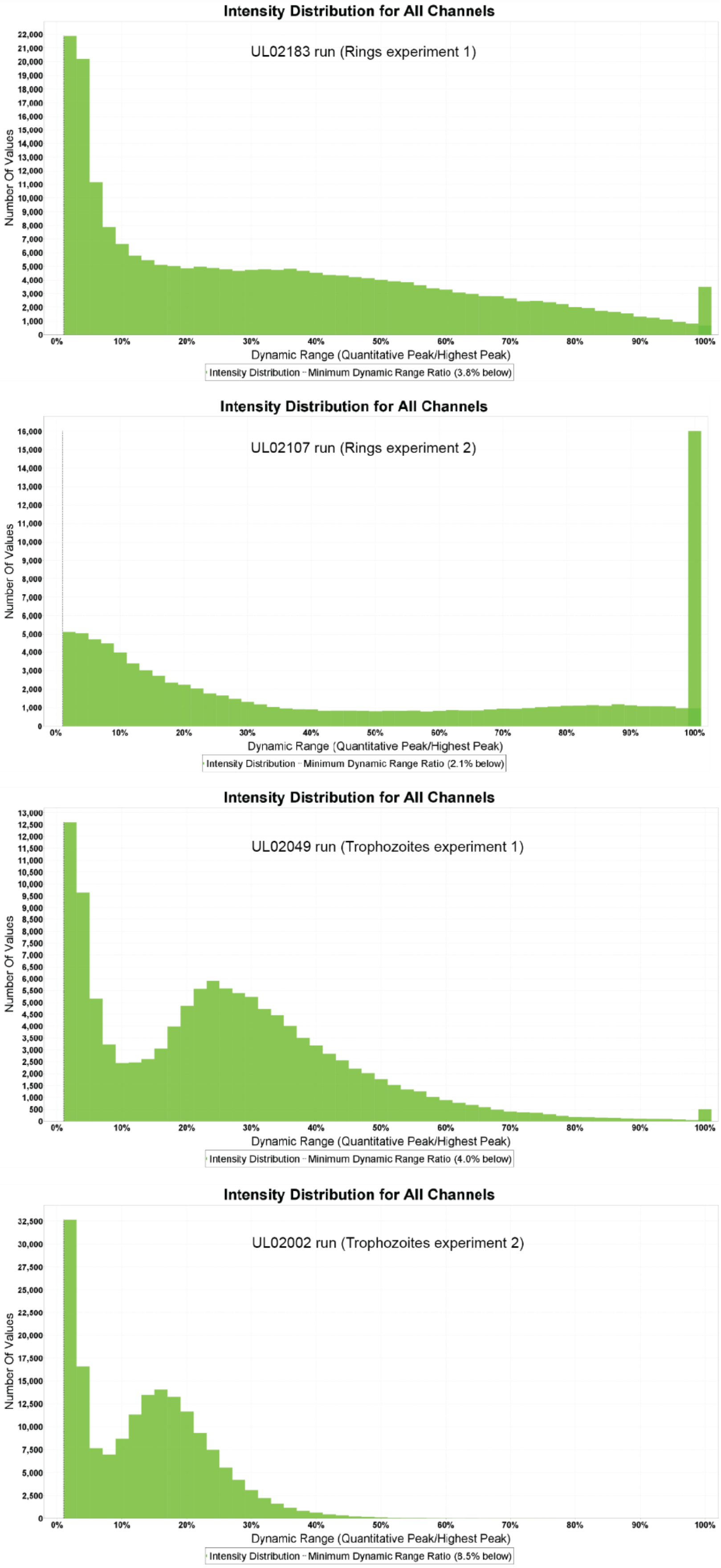

Supplementary Fig. 10. Distribution of reporter ion intensities across the TMT channels for each of the four LC-MS/MS runs using Cam3.II^{R539T}, Cam3.II^{C580Y} and Cam3.II^{WT} K13 ring and trophozoite stage parasites in proteomic experiments. Related to Fig. 3.

Supplementary Figure 11

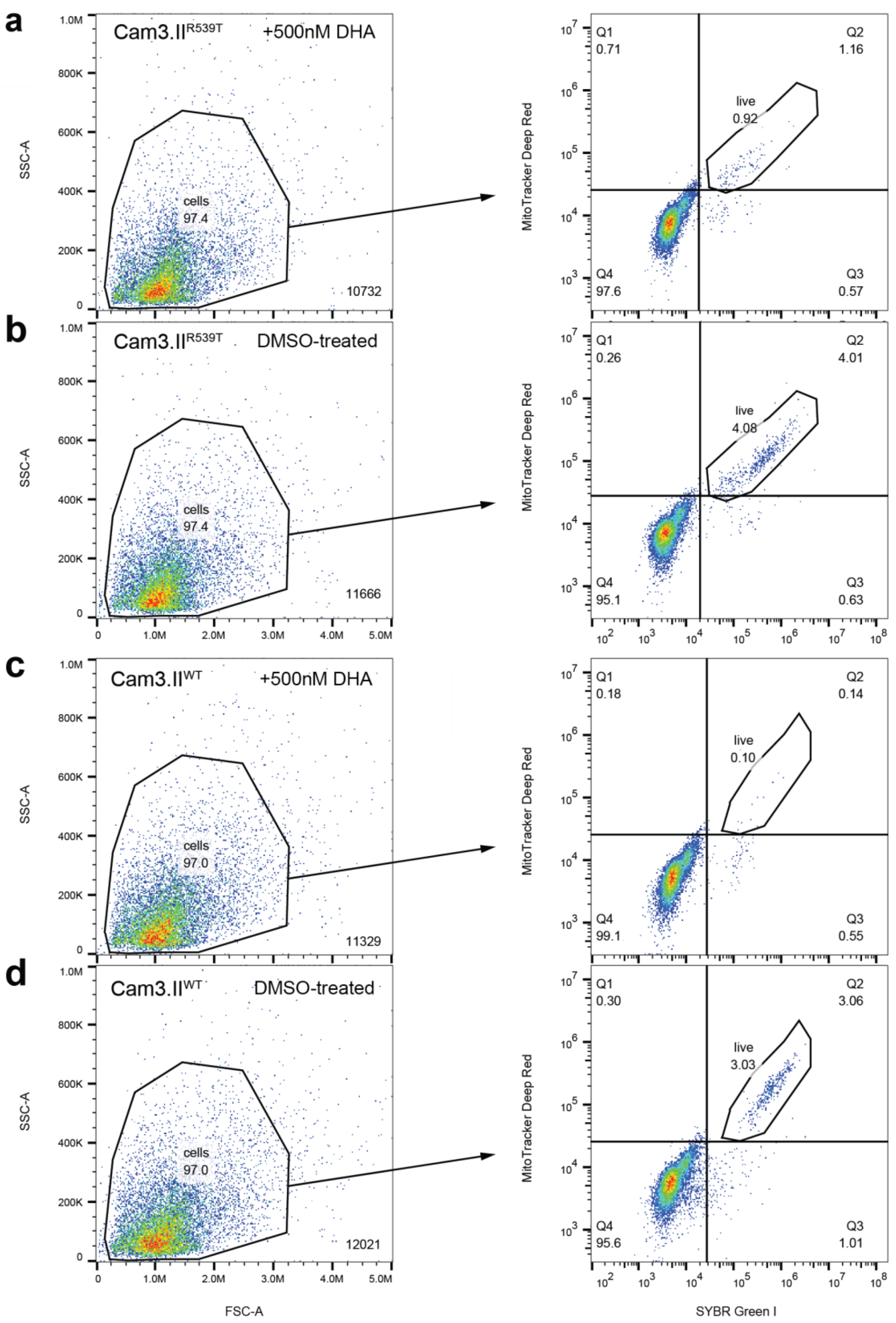

e

Sample	Count	Count of live	% live / total cells
Cam3.II^{R539T} + 500nM DHA	10732	96	0.92
Cam3.II^{R539T} + 0.1% DMSO	11666	463	4.08
Cam3.II^{WT} + 500nM DHA	11329	11	0.10
Cam3.II^{WT} + 0.1% DMSO	12021	353	3.03

Supplementary Figure 11. Flow cytometry pseudoplots showing the gating strategies for quantification of parasitemias in single and paired drug sensitivity assays. a-d, Between 10,000 and 15,000 events were counted per sample using an Accuri C6 flow cytometer. These panels show the gating strategy used to quantify viable parasites in dose-response assays. Cells were first gated by side and forward scatter (SSC-A and FSC-A) to remove extracellular debris. Within DHA-treated (**a, c**) or DMSO control-treated (**b, d**) cell populations, viable parasites positively stained by MitoTracker Deep Red and SYBR green I are shown in the upper right gate ("live") and are distinct from uninfected erythrocytes (MitoTracker Deep Red and SYBR green I negative; bottom left gates). **e**, Percent parasitemias are depicted as relative percentages of live parasite counts vs total erythrocyte counts. For each sample, percent survival was calculated as the ratio of parasitemia of the drug-treated vs the DMSO-treated control well. These data were used to determine IC values. Related to Fig. 5 and Supplementary Fig. 9.

Key Resources

REAGENT or RESOURCE	SOURCE	IDENTIFIER
Antibodies		
Mouse monoclonal anti-K13 E9	Ref. [4]	N/A
Chemicals, Peptides, and Recombinant Proteins		
D-Sorbitol	MP Biomedicals	Cat#ICN19474201
Percoll™ Centrifugation Media	GE Healthcare	Cat#17089101
Heparin, Sodium Salt, Porcine Intestinal Mucosa	Millipore Sigma	Cat#375095100KU
TRIzol™ Reagent	Invitrogen (Thermo Fisher Scientific)	Cat#15596018
Chloroform	Fisher	C/4960/17
GlycoBlue™ Coprecipitant	Invitrogen (Thermo Fisher Scientific)	Cat#AM9515
SuperScript™ II Reverse Transcriptase	Invitrogen (Thermo Fisher Scientific)	Cat#18064014
RNaseOUT™ Recombinant Ribonuclease Inhibitor	Invitrogen (Thermo Fisher Scientific)	Cat#10777019
Taq DNA Polymerase	New England BioLabs	Cat#M0273E
Saponin	Sigma-Aldrich	Cat#47036
cOmplete, EDTA-free Protease Inhibitor Cocktail	Roche (Sigma-Aldrich)	Cat#4693132001
5-Aminoallyl-dUTP (AA-dUTP)	Biotium	Cat#40020-1
Cyanine dyes Cy3 and Cy5	GE Healthcare	Cat#RPN5661
Trypsin from porcine pancreas	Sigma-Aldrich	Cat#T4799
$^{13}C_4$, ^{15}N-Aspartate	Cambridge Isotope	Cat#CNLM-544-H-PK
Dihydroartemisinin	AvaChem Scientific	Cat#1559
Atovaquone	Sigma-Aldrich	Cat#A7986
DSM265	Medicines for Malaria Venture (MMV)	CAS:1282041-94-4
Ribavirin	Sigma-Aldrich	Cat#R9644-10MG
Mycophenolic acid	Sigma-Aldrich	Cat#M3536-50MG
Fusidic acid	Sigma-Aldrich	Cat#F0881-1G
Immucilin-G	Ref. [5]	Vern L. Schramm, Albert Einstein College of Medicine, USA
Immucilin-H	Ref. [6]	Vern L. Schramm, Albert Einstein College of Medicine, USA

MitoTracker™ Deep Red FM	Invitrogen (Thermo Fisher Scientific)	Cat#M22426
SYBR™ Green I Nucleic Acid Gel Stain	Invitrogen (Thermo Fisher Scientific)	Cat#S7563
Critical Commercial Assays		
MycoAlert PLUS Mycoplasma Detection Kit	Lonza	Cat#LT07-707
MinElute PCR Purification Kit	QIAGEN	Cat#28006
Qubit RNA HS Assay Kit	Thermo Fisher Scientific	Cat#Q32852
Agilent 2X Hi-RPM Hybridization Buffer	Agilent	Cat#5190-0403
Pierce Direct IP Kit	Fisher Scientific	Cat#PI26148
TMTsixplex™ Isobaric Mass Tagging Kit	Thermo Scientific	Cat#90064
Deposited Data		
Raw and analyzed microarray data	This manuscript	GEO: GSE151189
Co-immunoprecipitation and mass spectrometry	Ref. [4]	Figure 3–Source Data 2
Proteomics LC-MS/MS	This manuscript	ProteomeXchange PXD019612
Metabolomics LC-MS/MS	This manuscript	NIH Metabolomics Workbench No. PR000864
P. falciparum 3D7 reference annotated transcriptome and proteome (release 46 and 28)<br>Biological process, components and functional activity: KEGG, GO, MPMP gene annotations	PlasmoDB	https://plasmodb.org/common/downloads/release-46/Pfalciparum3D7/
Experimental Models: Cell Lines		
Parasite strain: *P. falciparum* Dd2$^{bsm\ \mathrm{WT}}$	Ref. [7]	David A. Fidock, Columbia Uni., USA
Parasite strain: *P. falciparum* Dd2$^{bsm\ +\ \mathrm{R539T}}$	Ref. [7]	David A. Fidock, Columbia Uni., USA
Parasite strain: *P. falciparum* Dd2$^{bsm\ +\ \mathrm{C580Y}}$	Ref. [7]	David A. Fidock, Columbia Uni., USA
Parasite strain: *P. falciparum* Cam3.II$^{bsm\ +\ \mathrm{WT}}$	Ref. [7]	David A. Fidock, Columbia Uni., USA
Parasite strain: *P. falciparum* Cam3.II$^{\mathrm{R539T}}$	Ref. [7]	David A. Fidock, Columbia Uni., USA
Parasite strain: *P. falciparum* Cam3.II$^{bsm\ +\ \mathrm{C580Y}}$	Ref. [7]	David A. Fidock, Columbia Uni., USA
Oligonucleotides		

SMART-Random nonamers: AAGCAGTGGTATCAACGCAGAGTNNNNNNNNN	Sigma-Aldrich	N/A
SMART-Oligo-d(T)$_{30}$: AAGCAGTGGTATCAACGCAGAGTAC(T)$_{30}$VN	Sigma-Aldrich	N/A
TS-Oligo: AAGCAGTGGTATCAACGCAGAGTACGCrGrGrG	AITbiotech	N/A
Primer IIa: AAGCAGTGGTATCAACGCAGAGT	AITbiotech	N/A
Software and Algorithms		
R package Bioconductor and LIMMA (version 3.10.3)	Refs. [8-10]	RRID:SCR_010943; RRID:SCR_006442; https://www.bioconductor.org/
GenePix Pro version 6.0	Molecular Devices	RRID:SCR_010969
MeV (MultiExperiment Viewer) version 4.8.1	TM4	RRID:SCR_001915; https://sourceforge.net/projects/mev-tm4/
MetaboAnalystR version 3.0	Ref. [11]	RRID:SCR_016723; https://www.metaboanalyst.ca/
Proteome Discoverer™ version 2.1	Thermo Fisher Scientific	Cat#OPTON-30795; RRID:SCR_014477
Mascot version 2.6.0	Matrix Science Ltd.	RRID:SCR_014322; http://www.matrixscience.com/
UniProtKB-Swiss Prot database	UniProt	RRID:SCR_004426; https://www.uniprot.org/
Scaffold Q+ version 4.8.2	Proteome Software Inc.	RRID:SCR_014345; http://www.proteomesoftware.com/products/qplus/
FlowJo version 10	FlowJo LLC.	RRID:SCR_008520
GraphPad Prism version 7.03	GraphPad Software	RRID:SCR_002798
Other		
Custom 70-mer long oligo *P. falciparum* microarrays	This manuscript	GEO: GPL18893
Gold Seal™ Plain Microscope Slides	Thermo Fisher Scientific	Cat#3011-002
Agilent Hybridization Gasket Slide Kit	Agilent	Cat#G2534-60006
MACS CS Columns	Miltenyi Biotec, Inc.	Cat#130-041-305

Supplementary References

1. Foth, B. J., Zhang, N., Chaal, B. K., Sze, S. K., Preiser, P. R. & Bozdech, Z. Quantitative time-course profiling of parasite and host cell proteins in the human malaria parasite *Plasmodium falciparum*. *Mol. Cell. Proteomics* **10**, M110 006411 (2011).
2. MalariaGEN *Plasmodium falciparum* Community Project. Genomic epidemiology of artemisinin resistant malaria. *Elife* **5**, e08714 (2016).
3. Ocan, M. et al. K13-propeller gene polymorphisms in *Plasmodium falciparum* parasite population in malaria affected countries: a systematic review of prevalence and risk factors. *Malar. J.* **18**, 60 (2019).
4. Gnadig, N. F. et al. Insights into the intracellular localization, protein associations and artemisinin resistance properties of *Plasmodium falciparum* K13. *PLoS Pathog.* **16**, e1008482 (2020).
5. Ducati, R. G. et al. Genetic resistance to purine nucleoside phosphorylase inhibition in *Plasmodium falciparum*. *Proc. Natl. Acad. Sci. USA* **115**, 2114-2119 (2018).
6. Kicska, G. A., Tyler, P. C., Evans, G. B., Furneaux, R. H., Kim, K. & Schramm, V. L. Transition state analogue inhibitors of purine nucleoside phosphorylase from *Plasmodium falciparum*. *J. Biol. Chem.* **277**, 3219-3225 (2002).
7. Straimer, J. & et al. K13-propeller mutations confer artemisinin resistance in *Plasmodium falciparum* clinical isolates. *Science* **347**, 428-431 (2015).
8. Smyth, G. K. & Speed, T. Normalization of cDNA microarray data. *Methods* **31**, 265-273 (2003).
9. Huber, W. et al. Orchestrating high-throughput genomic analysis with Bioconductor. *Nat. Methods* **12**, 115-121 (2015).
10. Ritchie, M. E. et al. limma powers differential expression analyses for RNA-sequencing and microarray studies. *Nucleic Acids Res.* **43**, e47 (2015).
11. Chong, J. & Xia, J. MetaboAnalystR: an R package for flexible and reproducible analysis of metabolomics data. *Bioinformatics* **34**, 4313-4314 (2018).
