# Supplementary material for: Artemisinin-resistant K13 mutations rewire Plasmodium falciparum’s intra-erythrocytic metabolic program to enhance survival
Source: Nat Commun. 2021 Jan 22;12:530. doi: 10.1038/s41467-020-20805-w (PMC7822823; doi:10.1038/s41467-020-20805-w)
Supplement: Supplementary file 3 — Description of Additional Supplementary Files [file 41467_2020_20805_MOESM3_ESM.pdf]

Description of Additional Supplementary Files

File Name: Supplementary Data 1

Description: Up and down-regulated transcripts in Dd2 and Cam3.II K13 mutant lines relative to isogenic K13 wild-type lines, across the 48h intra-erythrocytic developmental cycle. List of 80 DE genes at late schizont and early ring stages. Up- or down-regulated transcripts are defined as having a P value < 0.05 based on a t-test, when comparing a K13 mutant against its isogenic wild-type counterpart. Related to Fig. 2 and Supplementary Fig. 2b.

File Name: Supplementary Data 2

Description: Up-regulated gene sets in Dd2 and Cam3.II K13 mutant lines, compared with K13 wild-type parasites, when assayed at a basal level without DHA exposure. Each sheet contains separate analyses for each sampling stage in a K13 mutant vs WT parasite. Total of 16 sheets containing pair-wise comparative results of upregulation of pathways in Cam3.IIR539T vs. Cam3.IIWT or Dd2R539T vs. Dd2WT or Dd2C580Y vs. Dd2WT parasites. Listed are the P values of hypergeometric testing for each independent analyses. No correction was made for multiple testing, as the goal of this exploratory work was to generate testable hypotheses. MPM: Malaria Parasite Metabolics; KEGG: Kyoto Encyclopedia of Genes and Genomes; GO: Gene Ontology; MF: molecular function; BP: biological process; CC: cellular component. Related to Fig. 2.

File Name: Supplementary Data 3

Description: List of 3186 proteins detected in at least one experimental LC-MS/MS run and differentially expressed (DE) proteins that were significantly up- and down-regulated in Cam3.II K13 mutants (R539T or C580Y) compared to the isogenic wild-type line harvested at ring or trophozoite stages. Up- or down-regulated proteins are defined as having a P-value < 0.05 based on a t-test on the log2 normalized peptide spectral intensities, when comparing a K13 mutant against its isogenic wild-type counterpart. Log2 fold-change (FC) and the standard deviation (SD) of the Log2 FC are listed for each protein. 8 proteins that map to multiple genes were filtered out from the list. Related to Fig. 3 and Supplementary Fig. 3.

File Name: Supplementary Data 4

Description: List of metabolites and their spectral peak areas detected in each experimental run for

Cam3.IIC580Y and Cam3.IIWT at the ring and trophozoite stages. Samples were either treated with DMSO vehicle control or with DHA at 70 nM or 350 nM for 3h before collection for LC-MS/MS. Related to Supplementary Fig. 5, and Fig. 3c and 4a.

File Name: Supplementary Data 5

Description: List of K13 co-immunoprecipitated proteins and their peptide counts in each sample. The 21 proteins were obtained after filtering for protein detected in at least 3 out of 6 experiments and at least 5 out of 13 samples. Gene descriptions and Gene Ontology (GO) components, functions and processes were annotated or computed from PlasmoDB v46. Related to Fig. 3d.

File Name: Supplementary Data 6

Description: List of gene sets from gene set enrichment analyses showing significant differential expression in the 3h and 6h post DHA-treated K13 WT and mutant Dd2 parasites relative to their respective DMSO controls (Nominal P

File Name: Supplementary Data 7

Description: List of functional pathways and genes that respond differently in the K13 mutant vs wild-type parasites upon exposure to 700 nM DHA. Total of 6 sheets. The first two sheets contain results of up-or down-regulation of genes in DHA-treated Dd2R539T vs. Dd2WT parasites at 3h and 6h post initiation of DHA treatment as shown in Fig. 4b. The last four sheets contain results of up-regulated genes in DHA-treated Dd2R539T vs. Dd2WT or Cam3.IIR539T vs. Cam3.IIWT parasites across the 48h sampling post initiation of DHA treatment. MPM: Malaria Parasite Metabolics; KEGG: Kyoto Encyclopedia of Genes and Genomes; GO: Gene Ontology; MF: molecular function; BP: biological process; CC: cellular component. Related to Fig. 4.

File Name: Supplementary Data 8

Description: IC50 and IC90 values of the individual dose response assays performed on Cam3.IIR539T and Cam3.IIWT parasites. Related to Fig. 5b.
