# Supplementary material for: Artemisinin-resistant K13 mutations rewire Plasmodium falciparum’s intra-erythrocytic metabolic program to enhance survival
Source: Nat Commun. 2021 Jan 22;12:530. doi: 10.1038/s41467-020-20805-w (PMC7822823; doi:10.1038/s41467-020-20805-w)
Supplement: Supplementary file 12 — Reporting Summary [file 41467_2020_20805_MOESM12_ESM.pdf]

nature research

Corresponding author(s): David A. Fidock

Last updated by author(s): Nov 25, 2020

Reporting Summary

Nature Research wishes to improve the reproducibility of the work that we publish. This form provides structure for consistency and transparency in reporting. For further information on Nature Research policies, see our Editorial Policies and the Editorial Policy Checklist.

Statistics

For all statistical analyses, confirm that the following items are present in the figure legend, table legend, main text, or Methods section.

n/a	Confirmed	
☐	☒	The exact sample size (*n*) for each experimental group/condition, given as a discrete number and unit of measurement
☐	☒	A statement on whether measurements were taken from distinct samples or whether the same sample was measured repeatedly
☐	☒	The statistical test(s) used AND whether they are one- or two-sided<br>*Only common tests should be described solely by name; describe more complex techniques in the Methods section.*
☐	☒	A description of all covariates tested
☐	☒	A description of any assumptions or corrections, such as tests of normality and adjustment for multiple comparisons
☐	☒	A full description of the statistical parameters including central tendency (e.g. means) or other basic estimates (e.g. regression coefficient) AND variation (e.g. standard deviation) or associated estimates of uncertainty (e.g. confidence intervals)
☐	☒	For null hypothesis testing, the test statistic (e.g. *F*, *t*, *r*) with confidence intervals, effect sizes, degrees of freedom and *P* value noted<br>*Give P values as exact values whenever suitable.*
☒	☐	For Bayesian analysis, information on the choice of priors and Markov chain Monte Carlo settings
☒	☐	For hierarchical and complex designs, identification of the appropriate level for tests and full reporting of outcomes
☒	☐	Estimates of effect sizes (e.g. Cohen's *d*, Pearson's *r*), indicating how they were calculated

Our web collection on statistics for biologists contains articles on many of the points above.

Software and code

Policy information about availability of computer code

Data collection	Transcriptomics: GenePix Pro 6.0 software (Molecular devices)<br>Proteomics and co-immunoprecipitation mass-spectrometry: Proteome Discoverer version 2.1 (Thermo Fisher Scientific), Mascot version 2.6.0 (Matrix Science Ltd.)<br>Metabolomics: The Metabolomic Analysis and Visualization Engine (MAVEN) software package (open source) version 8.0.3<br>Drug inhibition assays: FlowJo version 10 (FlowJo LLC)
Data analysis	Transcriptomics: R project version 2.14.2, Bioconductor and limma package version 3.10.3, MultiExperiment Viewer (MEV) version 4.8.1. (TM4)<br>Proteomics and co-immunoprecipitation mass-spectrometry: Scaffold Q+ version 4.8.2 (Proteome Software Inc.) using Protein Prophet algorithm version 5.0, STRING (Markov Clustering) version 11.0<br>Metabolomics: MetaboAnalyst 3.0 package in R<br>P. falciparum KEGG, GO, MPMP gene annotations and biological process, components and functional activity extracted from PlasmoDB release 46.<br>Drug inhibition assays: Prism version 7.03 (GraphPad)

For manuscripts utilizing custom algorithms or software that are central to the research but not yet described in published literature, software must be made available to editors and reviewers. We strongly encourage code deposition in a community repository (e.g. GitHub). See the Nature Research guidelines for submitting code & software for further information.

Data

Policy information about availability of data

All manuscripts must include a data availability statement. This statement should provide the following information, where applicable:

- Accession codes, unique identifiers, or web links for publicly available datasets
- A list of figures that have associated raw data
- A description of any restrictions on data availability

Microarray gene expression data are available in NCBI's Gene Expression Omnibus with the identifier GSE151189 (https://www.ncbi.nlm.nih.gov/geo/query/acc.cgi?acc=GSE151189). Data for mass spectrometry proteomics are available in ProteomeXchange Consortium (http://www.proteomexchange.org/) via the PRIDE repository database with the dataset identifier PXD019612 and doi:10.6019/PXD019612. LC-MS/MS metabolomics data are available at the NIH Common Fund's National Metabolomics Data Repository (NMDR) website, the Metabolomics Workbench (https://www.metabolomicsworkbench.org) with the project ID PR000864 and doi:10.21228/M80T2X. Datasets extracted from PlasmoDB (https://plasmodb.org), Kyoto Encyclopedia of Genes and Genomes (https://www.genome.jp/kegg/), Gene Ontology (http://geneontology.org/), Malaria Parasite Metabolic Pathways (https://mpmp.huji.ac.il/), and STRING (https://string-db.org/) can be accessed using these weblinks. The authors declare that all other data supporting the findings of this study are available within the paper and its supplementary information files. Source data for figures xxx are provided with the paper. Requests for resources and reagents should be directed to Dr. David Fidock (df2260@cumc.columbia.edu).

Field-specific reporting

Please select the one below that is the best fit for your research. If you are not sure, read the appropriate sections before making your selection.

- [x] Life sciences
- [ ] Behavioural & social sciences
- [ ] Ecological, evolutionary & environmental sciences

For a reference copy of the document with all sections, see nature.com/documents/nr-reporting-summary-flat.pdf

Life sciences study design

All studies must disclose on these points even when the disclosure is negative.

Sample size	Three independent biological experiments were performed per sample for transcriptomics and metabolomics LC-MS/MS to test for statistical significance. For transcriptomics, three samples provide sufficient sampling size as we also collected time course data across 6-7 time points over a 48h generation period per independent experiment, which allowed us to identify consistent changes across multiple time points at a specific developmental stage. For metabolomics, we collected three independent samples with up to three technical replicates for a total of seven samples per experimental group for examining stage-specific profiles. For proteomics mass-spectrometry, two independent sampling sets were collected per experimental group. This was the technical limits considering the large amount of biomaterial required per mass spectrometry run and the extremely small yield obtained from tightly synchronized early rings of P. falciparum. Three to five independent biological experiments were performed per sample for paired drug inhibition assays as the standard.
Data exclusions	Genes/protein/metabolites that had missing data in majority of samples were excluded during quality control checks, as described in methods section for each technique. Following these QC procedures, no data was further excluded from analyses.
Replication	During data collection, samples for K13 wild-type or mutant samples, and DHA or vehicle treated were always processed in parallel for every biological experiment and were carried out in consecutive generations of parasite growth to ensure reproducibility. Proteomic and metabolomic LC-MS/MS samples were multiplexed for each independent sampling set to ensure reproducibility in sample to sample comparisons. All attempts at replication were successful. For transcriptomics, samples were collected in three independent experiments for all parasite lines in the presence or absence of DHA, except for the DHA treatment of Cam3.IIR539T and Cam3.IIWT where one replicate each was performed. For proteomics, two independent sampling sets were collected per experimental group, except for Cam3.IIC580Y trophozoites that were sampled once. For metabolomics, three independent biological experiments were performed per sample for rings and one experiment for trophozoites. For paired drug inhibition assays, three to five independent biological experiments were performed per sample.
Randomization	Allocation of samples randomly into experimental groups was not relevant to our study as we sought to elucidate the role of K13 mutations in isogenic cells lines by carrying out supervised analyses.
Blinding	Investigators were blinded to group allocation during data processing. To avoid day-to-day bias in experimental data collection, samples processed for microarray hybridzations were randomized and microarray chips were barcoded. Samples for proteomics and metabolomics were blinded for our collaborators.

Reporting for specific materials, systems and methods

We require information from authors about some types of materials, experimental systems and methods used in many studies. Here, indicate whether each material, system or method listed is relevant to your study. If you are not sure if a list item applies to your research, read the appropriate section before selecting a response.

Materials & experimental systems

n/a	Involved in the study
☐	☒ Antibodies
☐	☒ Eukaryotic cell lines
☒	☐ Palaeontology and archaeology
☒	☐ Animals and other organisms
☒	☐ Human research participants
☒	☐ Clinical data
☒	☐ Dual use research of concern

Methods

n/a	Involved in the study
☒	☐ ChIP-seq
☐	☒ Flow cytometry
☒	☐ MRI-based neuroimaging

Antibodies

Antibodies used	Mouse monoclonal anti-K13 clone D3 and E9 (non-commercial source; Gnadig et al. PLoS Pathogens, 2020). 2.5ug of each antibody was used per 500ug of lysate per column, as noted in the Methods.
Validation	Antibodies were validated by testing against P. falciparum lines using co-immunoprecipitation mass-spectrometry, Western blots, immunofluorescence imaging, and cryo-EM.

Eukaryotic cell lines

Policy information about cell lines

Cell line source(s)	P. falciparum lines were generated in Straimer et al. Science, 2015.
Authentication	k13 gene editing of these lines was validated in Straimer et al. Science, 2015 and lines were subsequently routinely genotyped by Sanger sequencing of the k13 locus between experiments.
Mycoplasma contamination	All cell lines tested negative for Mycoplasma contamination.
Commonly misidentified lines (See ICLAC register)	No commonly misidentified lines were used in this study.

Flow Cytometry

Plots

Confirm that:

☒ The axis labels state the marker and fluorochrome used (e.g. CD4-FITC).

☒ The axis scales are clearly visible. Include numbers along axes only for bottom left plot of group (a 'group' is an analysis of identical markers).

☒ All plots are contour plots with outliers or pseudocolor plots.

☒ A numerical value for number of cells or percentage (with statistics) is provided.

Methodology

Sample preparation	P. falciparum parasite survival was assessed on an Accuri C6 flow cytometer (BD Biosciences) using SYBR Green I and MitoTracker Deep Red FM (Thermo Fisher Scientific) as stains for DNA and cell viability respectively.
Instrument	Accuri C6 (BD Biosciences) and HyperCyt autosampler (Intellicyt).
Software	FlowJo version 10 (FlowJo LLC.)
Cell population abundance	Flow cytometry was used to quantify percentage of live (fluorescence-labelled) parasites in a sample, which ranged from 0% to 8% parasitemia at various drug inhibitor concentrations. This method was not used for cell sorting.
Gating strategy	This gating method has been routinely performed to measure parasitemias, as earlier described (Straimer et al. Science, 2015). Flow counts were analyzed by first gating for red blood cells using FSC and SSC channels. Live parasites were determined as positive events for FL1 (SYBR Green) and FL4 (MitoTracker Deep Red) channels (upper right quadrant gate of plot). The percentage of live parasites was calculated as the number of live parasite events divided by the total red blood cell events for each sample. Supplementary figure 11 shows examples of Cam3.II R539T and wild-type parasites treated with DHA vs DMSO.

☒ Tick this box to confirm that a figure exemplifying the gating strategy is provided in the Supplementary Information.
